# Air pollution, dementia, and lifespan in the socio-economic gradient of aging: perspective on human aging for planning future experimental studies

**DOI:** 10.3389/fragi.2023.1273303

**Published:** 2023-11-13

**Authors:** Caleb E. Finch

**Affiliations:** Leonard Davis School of Gerontology and Dornsife College, University of Southern California, Los Angeles, CA, United States

**Keywords:** air pollution, developmental exposure, dementia, education, nematode, mouse

## Abstract

Air pollution (AirPoll) accelerates human aging, as assessed by increased adult mortality and earlier onset of cardiovascular diseases, and dementia. Socio-economic strata (SES) of wealth and education have parallel differences of mortality and these diseases. Children from impoverished homes differ in brain development at birth and in risk of early fat excess and hypertension. To further enhance the healthspan, biogerontologists may consider a wider range of environmental exposures from gestation through later life morbidity that comprise the Gero-Exposome. Experimental studies with rodents and nematodes document shared transcriptional responses to AirPoll. In rodents, AirPoll exposure activates gene systems for body-wide detoxification through Nrf2 and NFkB transcription factors that mediate multiple aging processes. Gestational environmental factors include maternal diet and exposure to AirPoll and cigarette smoke. Correspondingly, gestational exposure of mice to AirPoll increased adult body fat, impaired glucose clearance, and decreased adult neurogenesis in the hippocampus, a brain region damaged in dementia. Nematode larvae also respond to AirPoll with Alzheimer relevant responses. These experimental approaches could identify to interventions for expanded human health and longevity across SES gradients.

## 1 Introduction

This Perspective considers air pollution (AirPoll) as a global factor in excess mortality and risk of AD in framework of socio-economic strata (SES) Boing et al., 2022. The poor die younger with earlier onset of cardiovascular disease and AD. I propose that both AirPoll and SES accelerate human aging. Analyzing interactions of AirPoll and SES for convergent processes of aging could identify targets that expand the healthy lifespan for all of us.

Longevity has long been known for its low heritability (Finch and Tanzi, 1997; Finch and Loehlin, 1998). Twin studies consistently show heritability of lifespan of 20%–30% that may be even lower at later ages. The Swedish OCTO Twin Study had 12% heritability of age at death after 80 years (Johansson and Thorvaldsson, 2021). Decades of effort to identify longevity individual genes has identified few candidates, such as the alleles of ApoE and FOXO3A that are shared across human populations. This gap suggests the importance of population-specific genes (Caruso et al., 2022). Gene-environment (GxE) interactions for ApoE and FOXO3A have not been examined in depth. The wide variations of lifespan and later life health within human populations are mediated by myriad environmental factors and socio-economic strata (SES). Both are addressed by recent NIH programs (NIA, 2020, Health disparities; NIA, 2023, Exposome).

AirPoll is a major contributor to preventable (premature) deaths of 19 million, world-wide (Landrigan et al., 2018; Landrigan et al., 2022). Ambient PM2.5 represents inhalable particulate matter (PM) of 2.5 micron diameter or smaller, as measured daily by the Environmental Protection Agency across the United States. PM2.5 composition may vary widely at any place depending, for example, on traffic density, industrial activity, and seasonal fires. Despite this heterogeneity, the global associations of PM2.5 with many diseases and mortality show the same linearity and scale: no level of PM2.5 is safe, like cigarette smoke. The impact of PM2.5 extends to gestational exposures, as described below for the brain (Dickerson et al., 2023). Prenatal exposures by SES may differ widely between and within countries for maternal diet, drugs, and smoking exposure.

## 2 Air pollution and SES are global environmental factors in aging

**FIGURE 1:**
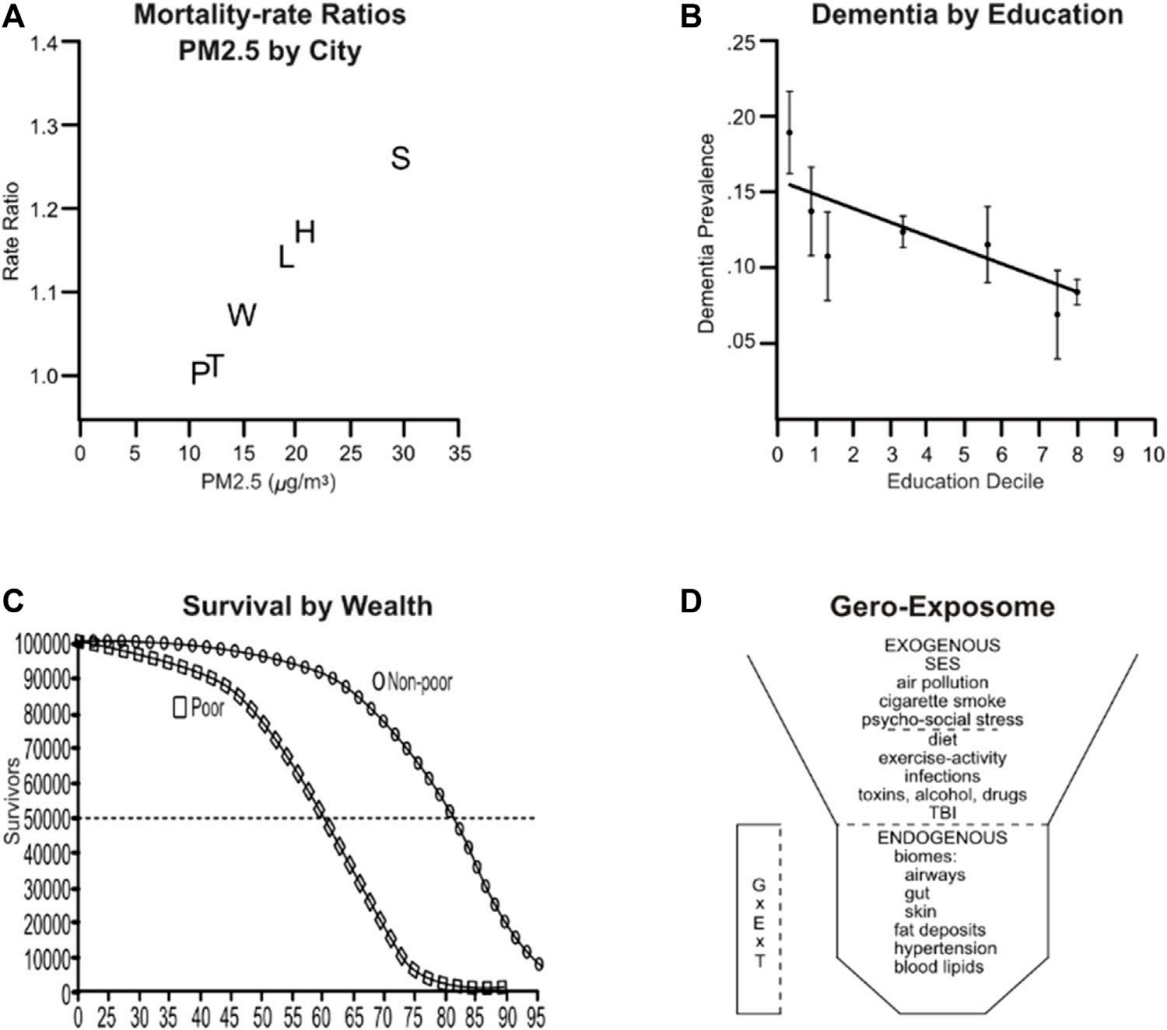
Air pollution enhancement for risk of mortality and AD are modified by SES. **(A)** AirPoll and mortality rates from the ‘Six Cities Study’ of air pollution and mortality. White subjects, age 25–74, were enrolled in 1974 and followed to 1991. City names: P, Portage WI; T, Topeka KA; H, Harriman TN; Q, Watertown MA; L, St. Louis MO; S, Steubenville OH. Redrawn from Dockery et al. (1993). **(B)** AD prevalence by education decile in the United States ; standardized for age and gender, ±95% CI. Redrawn from Arakapis et al. (2021). **(C)** Survival based on data from the US-wide National Health and Nutrition Examination Survey (NHANES). Redrawn from Crimmins et al. (2009). **(D)** The Gero-Exposome with exogenous and endogenous components. Abbreviations: GxExT, Interactions of gene by environment over age and time; SES, socioeconomic status; TBI, traumatic brain injury. Redrawn from Finch and Haghani 2021, Finch and Kulminski 2019.

The 1993 benchmark ‘Six Cities Study’ (Dockery et al., 1993) showed strong associations of adult mortality over 3-fold levels of urban PM2.5 (Figure 1A). Six Cities pioneered in longitudinal exposures and in considering SES and smoking. Nonsmokers had lower mortality in cities across the low to high extremes of PM2.5 (OR, 1.19) than current smokers (OR 1.32). Body mass index was associated with smaller risk (OR 1.08). After ‘controlling for’ education and for cigarette smoking as mortality risks, associations of PM2.5 with mortality remained strongly significant. We now know that smoking synergizes with PM2.5 for super-additive increased risk of lung cancer and cardiovascular disease (Turner et al., 2014; Forman and Finch, 2018). These finding have been replicated in multiple populations.

AD risk also varies inversely with SES in the United States (Figure 1B), England (Arakapis et al., 2021), Finland (Korhonen et al., 2023), among other economically developed countries. Moreover, longevity parallels this pattern: United States survival curves for ‘poor vs. non-poor’ are shifted more than 10 years (Crimmins et al., 2009; Chetty et al., 2016) (Figure 1C). DNA me also varies by SES (Faul et al., 2023). In effect, the poor age faster, with earlier onset of obesity, cardiovascular diseases, and AD, all following SES gradients.

AirPoll PM2.5 elevations are associated with accelerated cognitive decline and AD in multiple cohorts from different populations (Cacciottolo et al., 2017; Chen et al., 2020; De Looze et al., 2023; Franz et al., 2023; Petkus et al., 2021; Wang et al., 2023; Yuan et al., 2023). However, these cohorts were not designed for SES representation. This gap was recently addressed with the US-wide MESA Air pollution Study of 4,392 adults (Wang et al., 2023). Followed longitudinally for a decade since age 62, lower SES had stronger cognitive associations with specific chemical components of PM2.5: elementary carbon (EC, tailpipe) and silicon (Si, non-tailpipe dust) were each associated with decrease of particular cognitive processes. Some low SES populations also incur higher exposure to cigarette smoke and PM2.5. Gene-environment (GxE) interactions may be anticipated. ApoE4, the AD risk allele, increased the risk of AD for high PM2.5 in some populations (Cacciottolo et al., 2017; Franz et al., 2023; Kulick et al., 2021; Christensen et al., 2023). ApoE4 and neighboring genes on chromosome 19q13.3 elevate blood cholesterol and increases risk of heart attack (Nazarian et al., 2022), ApoE4 did not further increase in smokers (Holmes et al., 2014).

The multitude of environmental factors in AD can be conceptually organized in the ‘Gero Exposome’ (Figure 1D), comprised of *exogenous* vs. *endogenous* factors (Finch and Kulminski, 2019; Finch and Haghani, 2021). Gene-environment factors must operate across the lifespan, including early development (GxExT). In this schema, SES and PM2.5l are general exogenous factors not controlled by individual life-style choices. Diet and smoking are individual exogenous factors, while body fat is an individual endogenous factor. Links of PM2.5 to cardiovascular disease for body fat and smoking are better defined than for AD. In the Los Angeles Children’s Health Study, prenatal exposure to elevated PM2.5 increased carotid stiffness and systolic blood pressure at age 11 (Breton et al., 2016).

Brain development is also vulnerable in lower SES households, shown by MRI studies (Spann et al., 2020; McKinnon et al., 2023; Morgan et al., 2023; Thomas and Coecke, 2023). For example, lower SES neonates had larger volumes of several frontal cortex subregions in association with poorer language skill by age 2 years (Spann et al., 2020). A meta-analysis expanded these findings of brain region specificity to SES, with enlarged superior temporal gyri and hippocampus in lower SES children (Vannucci et al., 2023). Conversely, lower SES showed smaller right-side fronto-parietal cortex (Spann et al., 2020). These early findings are hard to compare between studies because of small sample size, different imaging measures, and undefined heterogeneity of SES environments (Thomas and Coecke, 2023). Caveats accepted: because these forebrain regions are also affected by AD at later ages, their SES sensitivity may contribute to the earlier onset of AD.

## 3 Experimental models

Experimental models for AirPoll neurotoxicity are well developed, based on pioneering studies of Calderón-Garcidueñas (2002) and Block (Block et al., 2004; Levesque et al., 2011) that showed rodent brain inflammation and increased amyloid peptides from exposure to ambient urban air and diesel exhaust, respectively. Many labs study how PM2.5 and subfractions can accelerate diseases of the brain and cerebral arteries in rodent models. Nematodes also respond toPM2.5 subfractions, as noted below.

**FIGURE 2:**
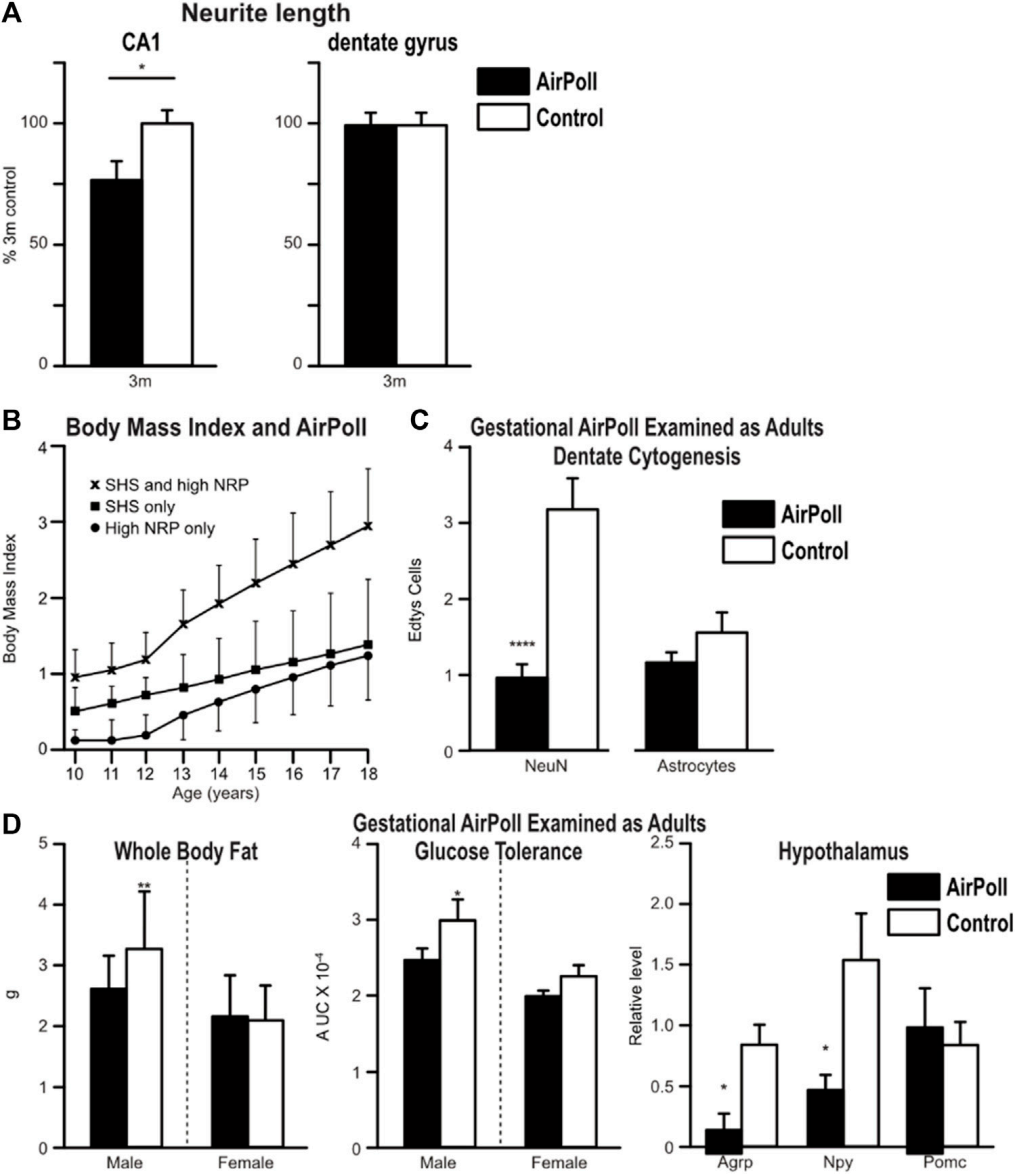
Toxicity of air pollution particles. **(A)** Adult mouse brain responses to AirPoll by 3 month old wild-type mice (C57/BL6) with 8 weeks exposure to AirPoll-nPM. In the hippocampus, neurons of the CA1 stratum oriens had neurites shortened by 30% by nPM. However, in the neighboring dentate gyrus, neurite length was unchanged. Redrawn from Woodward et al. (2017b). **(B)** Adolescent body fat (body mass index, BMI) was increased by exposure to AirPoll, measured as near roadway (NRP) and second-hand cigarette smoke (SHS) from adult smokers. Los Angeles Children Study. Redrawn from McConnell et al. (2015). **(C)** Gestational exposure of mice to nPM impaired adult dentate gyrus neurogenesis, assayed by incorporation uracil nucleotide EdU. Neuronal generation was decreased >50%, whereas astrocyte generation was not altered. Redrawn from Woodward et al. (2018). **(D)** Gestational exposure also increased adult body fat and impaired glucose clearance, redrawn from Woodward et al. (2019), and altered hypothalamic peptides, redrawn from Haghani et al. (2020a).

Our rodent exposure studies have used several subfractions of PM2.5 in the ultrafine size class, PM0.2, which may penetrate more deeply into airways than the PM2.5 (Finch, 2018, p.51). With technology designed by Costas Sioutas at USC, we exposed rodents to a re-aerosolized nano-sized subfraction of PM2.5 from ambient Los Angeles roadway air (nPM), or from diesel exhaust particles (DEP) at controlled density for 8 weeks (5 h/d, 5 days/wk). The nPM are an aqueous solubilized subfraction that lacks polyaromatic hydrocarbons (PAH). Exposure to nPM caused activated glial, and induced inflammation in cerebral cortex, nPM caused 50% increase of microglial CD14 and CD68 and astrocytic GFAP, with 50% higher inflammatory cytokines IL-1α and TNFα. Transcriptional responses include induction of genes for detoxification and oxidative repair via transcription factors Nrf2 and NfkB (Morgan et al., 2011; Zhang et al., 2012; Chepelev et al., 2013; Haghani et al., 2020b; Haghani et al., 2021). These AirPoll pathways are also shared with systemic oxidative aging processes recognized as a ‘Hallmark of Aging’ (Schmidlin et al., 2019; Maldonaldo et al., 2023). Moreover, in the hippocampus, nPM caused selective loss, paralleling AD (Woodward et al., 2017a). Neurites were shortened in the CA1 neuronal layer (Figure 2A), which is damaged in AD; in contrast, neurite length was not altered in the adjacent dentate gyrus, which is resistant to AD. The GluA1 glutamate receptor subunit was decreased (Woodward et al. 2017a). White matter myelin was also damaged (Huuskonen et al., 2021; Lamorie-Foote et al., 2023).

Unexpectedly the neurotoxicity of locally obtained nPM decreased after 2018 (Zhang et al., 2021). To obtain reliable responses of neurotoxicity, we have switched to DEP for rodent exposures which replicate most of our prior findings with nPM (Shkirkova et al., 2022). Standardized DEP are availability from the National Institute of Science and Technology (NIST SRM 2975), collected from a single diesel engine. These DEP have some PAHs, unlike nPM, and fewer redox-active metals are also much lower than ambient Pm2.5 (Farahani et al., 2021; Zhang et al., 2021). The NIST-DEP may stimulate other labs to join this growing field with expectation of expanded verifiability of studies which is required for mechanistic studies. A historical precedent may be the introduction of “Kentucky reference cigarettes” developed decades ago, which enhanced cancer research replicability (Jacard et al., 2019).

Because exposure to AirPoll andcigarette smoke can begin prenatally with maternal exposure, we must analyze the impact of air pollution across the entire human life course. This concept was stimulated by the elegant Los Angeles Children Study, which examined the body mass index (BMI) of adolescents in households that differed by distance from major roadways and adult smoking (McConnell et al., 2015) (Figure 2B). Roadway closeness and adult smoking each increased childrens’ BMI; again, the combination was super-additive. We do not know the molecular basis for these multiple synergies of AirPoll neurotoxicity, noted above for lung cancer. Lower SES populations also have earlier and higher BMI, more cardiometabolic diseases, and more exposure to cigarette smoke.

We developed models for gestational exposure of rodents to nPM which altered adult metabolism. The first study began exposure 7 weeks before mating, which exposed the maturing primary oocyte before fertilization (Davis et al., 2013). Subsequent studies exposed mice only during gestation with similar results (Woodward et al., 2018; Woodward et al., 2019). We tentatively conclude that the major developmental vulnerability to AirPoll arises post fertilization. Further studies could define if the preimplantation zygote is vulnerable to AirPoll. Gestational exposure to nPM of wild-type mice caused multiple adult impairments. The hippocampus had impaired adult neuronal stem cell proliferation (Figure 2C) (Woodward et al., 2018). Adult mice were also fatter with impaired glucose clearance (Figure 2D). The hypothalamic metabolic axis was also damaged with >50% decreases of the neuropeptides AGRP, NPY, and POMC (Figure 2D). These findings suggest that the elevated BMI of adolescents (Figure 2B) may include gestational exposure to inhaled toxins of AirPoll and cigarette smoke.

The nematode *C. elegans* is also sensitive to AirPoll, using nPM (Haghani et al., 2019). Exposure of Stage 1 larvae to non-lethal concentrations decreased adult size and modified AD-related expression of *sel-12*, an amyloid-processing gene (Haghani et al., 2019) and increased amyloid protein aggregates (Garcia Manriquez et al., 2023). Again, these responses involve the *Nrf2* anti-oxidant gene system (*skn-1* in nematodes).

## 4 Discussion

Four decades ago, the pioneering Six Cities Study associated AirPoll and mortality with SES differences. We now know AirPoll accelerates many aging processes with mechanisms that are shared with cardiovascular disease and AD. Prenatal exposure to maternal inhalation may the first critical phase for AirPoll impact on the brain and arteries.

A further prenatal phase merits consideration, that the egg we came from was formed in our mothers’ ovary before her birth, as known to embryologists for a century. While most oocytes are as old as our mother, some *de novo* oogenesis may occur (Porras-Gómez and Moreno-Mendoza, 2017; Nagamatsu, 2023). Finch and Loehlin. (1998) proposed the ‘pre-zygotic hypothesis’ for potential for multigenerational environmental effects on brain function mediated by environmental impact on the oocyte before fertilization. Multigenerational persistence of gestational toxicity is documented in mice for maternal lead, where the third generation after gestational exposure of mice had altered locomotion and blood corticosterone (Sobolewski et al., 2018; Sobolewski et al., 2020). The decrease of ovarian follicles by prenatal exposure to diesel exhaust (Ogliari et al., 2013) warrants analysis for potential multigenerational impact of AirPoll.

Major issues remain open. We do not know how maternal inhalation of PM0.2 can so profoundly impact systemic metabolism. Some particles may pass lung into blood,but would then encounter the robust placental barrier (Finch, 2018, p.66). Future studies may define the maternal and fetal proteome and lipidome response to AirPoll. The findings of Wang et al (2023) discussed above anticipate more chemical specification of AirPoll components on artery and brain development. Shared factors in the Cardiovascular and AD Exposomes are likely to differ with SES. We may anticipate new interventions to expand the health span for all SES as mechanistic pathways becomeresolved for GxExT interactions.

## Data Availability

The original contributions presented in the study are included in the article/Supplementary Materials, further inquiries can be directed to the corresponding author.
